# Measuring Internet Meme engagement and individual differences: a novel scale and its correlates

**DOI:** 10.23889/ijpds.v8i3.2276

**Published:** 2023-09-11

**Authors:** Giovanni Schiazza

**Affiliations:** 1 Mixed Reality Lab, University of Nottingham

## Introduction & Background

Internet Memes (IMs) are social, digital artefacts that act as information vectors on social networking sites. Memetic scholarly literature has mainly focused on analysing IMs content with mixed methods. However, little scholarly attention has been devoted to exploring the relationships between IMs and users through survey methodologies.

Users engage with IMs in many ways, but scholarly literature lacks studies on Individual Differences (ID) that might make users more or less prone to engage with them. The results suggest that certain psychological factors may affect IM engagement.

## Objectives & Approach

This study examines how individual determinants relate to general and political internet meme engagement. An exploratory survey design is employed on an online sample of 472 participants.

To measure meme engagement, we develop a novel scale by asking participants how likely (1-7) they are to exhibit certain behaviours (liking, commenting, sharing on account, tagging or sending privately to someone) on general memes and politically-centred ones. The novel scales’ feasibility is tested, achieving good internal reliability (α>0.7) and a good fit in confirmatory factor analysis.

The survey included validated measures of Fear of Missing Out (FOMO), Conspiracy Belief (CB), personality traits (Big-5), Bullshit Receptivity (BR), Critical Reflection Test (CRT) and an adaptation of Social Network Intensity (SNI). All the measures employed achieved good internal consistency (α>0.7).

The study thematically groups the measures related to IMs (engagement, familiarity and attitude), social media (FOMO, SNI), cognitive style (CRT, BR, CB), personality (Big-5) and socio-demographics (age, gender, education, ethnicity, nationality, ideology).

## Relevance to Digital Footprints

With increasing interest and research being done on computational analysis of social media and its phenomena, there is a need for survey research to explore connections between IDs and user behaviour through using a mix of validated and novel ad-hoc measures.

User interaction with internet memes creates a data trail that can be used to infer several individual determinants through machine learning techniques. However, further psychological research is needed to assess and underpin the linkages between IDs and IM engagement before inferring IDs on large datasets.

## Results

Bivariate correlations reveal that young age, extraversion, neuroticism, SNI, FOMO, BR and CB are positively associated with internet meme engagement regardless of content. Further, t-tests of dependent correlations show that age, FOMO and ideology differ significantly in their correlations between general vs political meme engagement.

Engagement with political IMs is slightly higher in people with left-leaning ideology and lower levels of conscientiousness. A positive attitude towards IMs correlates with a marginally higher openness to experience and left ideology while strongly correlating with younger age.

Interestingly, traditional measures of cognitive style do not correlate with IM engagement, while lower education weakly correlates with self-assessed IM familiarity. Only BR positively correlates with IM engagement, suggesting that gullibility plays a role in engagement.

In hierarchical regressions, thematic groupings predict differences in variance shares in IM engagement. More overall variance (+10%) is explained in the general IM engagement scale compared to political IM engagement; socio-demo and cognitive account for less variance in political IMs. In comparison, personality accounts for slightly more variance in political IM engagement. This analysis further supports different relational structures of IDs when looking at general vs political IM engagement.

## Conclusions & Implications

Study results suggest a difference in the pattern of engagement depending on the content of IMs and participants’ IDs.

This exploratory study shows early signs that IDs play a role in IM engagement, opening avenues for further survey research and identifying which specific IDs might be interesting to investigate when performing this type of research.

This study’s findings help researchers and users better understand the heterogeneous world of IM topical engagement by highlighting what IDs might make users more prone to engage with these social, digital artefacts.

